# Crystal Structure of Cytochrome *c*_L_ from the Aquatic Methylotrophic Bacterium *Methylophaga aminisulfidivorans* MP_T_

**DOI:** 10.4014/jmb.2006.06029

**Published:** 2020-05-20

**Authors:** Suparna Ghosh, Immanuel Dhanasingh,, Jaewon Ryu, Si Wouk Kim, Sung Haeng Lee

**Affiliations:** 1 Department of Cellular and Molecular Medicine, Chosun University School of Medicine, Gwangju 61452, Republic of Korea; 2 Department of Biomedical Sciences, Graduate School of Chosun University, Gwangju 61452, Republic of Korea; 3 Department of Environmental Engineering, Chosun University, Gwangju 61452, Republic of Korea; 4 Department of Energy Convergence, Graduate School of Chosun University, Gwangju 61452, Republic of Korea

**Keywords:** Methylotroph, electron transfer, methanol dehydrogenase complex, methanol oxidation, Cytochrome *c*_L_, *Methylophaga aminisulfidivorans* MP^T^

## Abstract

Cytochrome *c*_L_ (Cyt*c*_L_) is an essential protein in the process of methanol oxidation in methylotrophs. It receives an electron from the pyrroloquinoline quinone (PQQ) cofactor of methanol dehydrogenase (MDH) to produce formaldehyde. The direct electron transfer mechanism between Cyt*c*_L_ and MDH remains unknown due to the lack of structural information. To help gain a better understanding of the mechanism, we determined the first crystal structure of heme *c* containing Cyt*c*_L_ from the aquatic methylotrophic bacterium *Methylophaga aminisulfidivorans* MP^T^ at 2.13 Å resolution. The crystal structure of *Ma*-Cyt*c*_L_ revealed its unique features compared to those of the terrestrial homologues. Apart from Fe in heme, three additional metal ion binding sites for Na^+^, Ca^+^, and Fe^2+^ were found, wherein the ions mostly formed coordination bonds with the amino acid residues on the loop (G93-Y111) that interacts with heme. Therefore, these ions seemed to enhance the stability of heme insertion by increasing the loop's steadiness. The basic N-terminal end, together with helix α4 and loop (G126 to Y136), contributed positive charge to the region. In contrast, the acidic C-terminal end provided a negatively charged surface, yielding several electrostatic contact points with partner proteins for electron transfer. These exceptional features of *Ma*-Cyt*c*_L_, along with the structural information of MDH, led us to hypothesize the need for an adapter protein bridging MDH to Cyt*c*_L_ within appropriate proximity for electron transfer. With this knowledge in mind, the methanol oxidation complex reconstitution in vitro could be utilized to produce metabolic intermediates at the industry level.

## Introduction

**Fig. 1:**
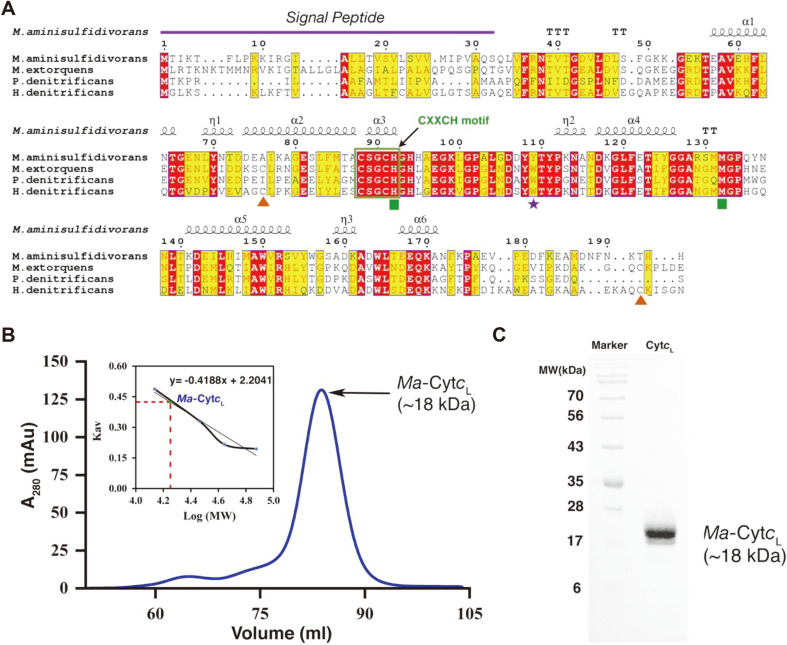
Sequence alignment of Cyt*c*L from *Methylophaga aminisulfidivorans* MPT with close structural homologs and oligomeric state determination.(**A**) Sequence alignment. The alignment of the sequence of *M. aminisulfidivorans* MPT with the sequences of its close structural homologs was performed using the T-Coffee web server, followed by rendering using Espript 3.0. The first 32 residues, predicted to be the signal peptide, are marked with a purple line. The identical conserved residues are shown in red and similar residues are shown in yellow. The sequence containing the conserved heme-binding CXXCH motif is boxed green. The residues corresponding to the C-terminal disulfide bridge in other homologs are indicated by the orange triangle; the axial ligands M132 and H92 residue are shown by the green square. The evolutionarily mutated residue Tyrosine Y109 of *Ma*-CytcL, in place of tryptophan in other homologs, is marked with a purple star. (**B**) Oligomeric state determination using size exclusion chromatography. The final purified *Ma*-Cyt*c*L was run through a size exclusion column, with its molecular weight shown to be 17.67 kDa. The standard plot of k_av_ *vs*. log Mr was used to determine the molecular weight based on peak elution. (**C**) The final purified protein was run through an 15% SDS PAGE gel, which shows a band at an approximate molecular weight of 18 kDa.

Methylotrophic bacteria utilize methanol as their single carbon source of energy by oxidizing it to formaldehyde (Fig. 1) [1]. Methylotrophs are of great interest in the study of the biogeochemical cycling of methanol as well as the commercial production of complex polymers such as urea-formaldehyde resin and polyoxymethylene plastics [2]. The oxidation of methanol for energy in methylotrophic bacteria is carried out under the control of the methanol oxidizing (*mox*) operon, which consists of genes such as *mxaF*, *mxaI*, *mxaJ*, *mxaG, mxaR,* and *mxaS*, encoding α- and β- subunits of methanol dehydrogenase (MDH), MxaJ, cytochrome *c*_L_ (Cyt*c*_L_), MxaR, and MxaS, respectively (Fig. S1A) [3]. MDH comprises two subunits (α and β) and a cofactor pyrroloquinoline quinone (PQQ), which mobilizes two electrons from methanol, thereby converting it to formaldehyde [4]. The two electrons are subsequently transferred from PQQ of MDH to the heme of cytochrome *c*_L_ through an unknown mechanism, followed by its sequential transfer to Cyt*c*H, cytochrome *c* oxidase, and finally to ATP synthase in the cell membrane for the generation of ATP molecules (Fig. S1B) [5-8].

The electron transfer from MDH to Cyt*c*_L_ requires complex formation between these two proteins. Previous studies have proposed an interaction between the basic protein MDH and the acidic protein Cyt*c*_L_ for expedient electron transfer [6, 9, 10]. This complex formation is perplexing since it has never been isolated as a stable complex for crystallization, leading to the unavailability of a structure of the same till date. The docked protein complex [6] showed that the distance between the PQQ in the active site of MDH and the heme of Cyt*c*_L_ was approximately 20 Å, which was beyond the ideal electron jump distance (~14 Å) [11]. Furthermore, it was found that salt inhibits the initial complex formation and the subsequent electron transfer, and the inhibition is proportional to the ionic strength of the medium [6, 10]. These observations suggest a novel role for the other protein components of *mox* operon, such as MxaJ, MxaR, and MxaS, in aiding the electron transfer between MDH and Cyt*c*_L_ [12, 13], especially in bacteria living in seawater (3.5% NaCl) (Fig. S1B) [12]. It underscores the need for further research in order to understand the strategies adapted by marine bacteria to circumvent the high salinity conditions which have an inhibitory effect on electron transfer, the latter being an essential step in the oxidation of methanol to obtain energy.

The marine bacterium *Methylophaga aminisulfidivorans* MP^T^ (*Ma*MP^T^), which was isolated from the sea waters of Mokpo, South Korea, was classified as a neutrophilic, moderately halophilic, and vitamin B12-independent facultative methylotroph [14, 15]. We had already reported the structures of the *mox* operon proteins *Ma*-MDH [16, 17] and *Ma*-MxaJ [12, 13], which are expressed in the periplasmic region of the *Ma*MP^T^. We had previously shown that the overall interaction between β-subunits and α-subunits of *Ma*-MDH was stronger than that of terrestrial homologs, providing the structural integrity to *Ma*-MDH in aquatic environments [16]. The floor of its active site was formed of tryptophan residues, while the ceiling was formed by the disulfide bridge (PDB:5XM3). The presence of two extra tryptophan residues in *Ma*-MDH resulted in a stronger hydrophobic core compared to the terrestrial homologs. In addition, the typical Ca^2+^ binding site near PQQ in terrestrial bacteria [18, 19] was replaced with Mg^2+^, which is abundant in the marine bacterium *Ma*MP^T^ [20]. While the MDH of terrestrial homologs was basic, the marine *Ma*-MDH had unique acidic residues at the interface of the *Ma*-MDH hetero- tetramer (α_2_β_2_), suggesting that the basic residues of its binding partner, which could be either *Ma*-MxaJ or *Ma*- Cyt*c*_L_, might contribute towards complex formation [16].

The novel fold in *Ma*-MxaJ was shown to contain the ‘bi-lobate’ folding architecture found in periplasmic binding proteins (PDB:5SV6) [12]. A distinctive structural feature of *Ma*-MxaJ was the presence of an acidic cavity at the interface of the two domains. This provided a more favorable environment for the interaction with proteins, suggesting that it could be a plausible binding partner for either *Ma*-MDH or *Ma*-Cyt*c*_L_ [12]. The characteristic basic loop L8 between helices α5 and α6 in *the Ma*-MxaJ structure was expected to bind to the acidic MDH hetero-tetramer [12]. The structure elucidation of *Ma*-Cyt*c*_L_ would be a crucial step in gaining a deeper insight into the overall mechanism of the methanol oxidation system and the electron transfer between the *mox* gene cluster proteins in a marine bacterium. Here, we report the first crystal structure of Cyt*c*_L_, the electron acceptor from the marine bacterium *M. aminisulfidivorans* MP^T^, and postulate the plausible mechanism of electron transfer.

The sequence of *Ma*-Cyt*c*_L_ from *Ma*MP^T^ showed high homology with the structures of Cyt*c*_L_s from soil bacteria including *Methylobacterium extorquens* (*Me*-Cyt*c*_L_; PDB: 2D0W)[6], *Hyphomicrobium denitrificans* (*Hd*-Cyt*c*_L_; PDB: 2C8S) [7] and *Paracoccus denitrificans* (*Pd*-Cyt*c*_L_; PDB: 2GC4) [21], with the sequence identities ranging from 50-60%. The cytochrome *c* fold included four α-helices protecting the heme, followed by three more α- helices around them. Although the core structural elements and the sequence of Cyt*c*_L_s were identical to the cytochrome domain present in proteins such as quino-hemoprotein alcohol dehydrogenase, the N- and C- terminal residues of Cyt*c*_L_ were unique [6]. Indeed, *Ma*-Cyt*c*_L_ had basic and acidic residues at the N-terminus and C-terminus ends, which might have a role to play in protein binding and electron transfer.

Here, we determined the structure of *Ma*-Cyt*c*_L_ at 2.13 Å resolution and identified the structural features, which distinguish it from its homologs in terrestrial methylotrophs. The distinctive structural features included the flexible ends of N- and C-terminal providing positively and negatively charged surfaces, respectively, thereby increasing the number of potential contact points with the binding partner. Furthermore, the efficient strategy of utilizing metals for enhancing the steadiness of the loop in the heme-binding motif was intriguing. Based on the exclusive structural features of *Ma*-Cyt*c*_L_ compared to its terrestrial homologs, we discuss its possible interaction with the tertiary proteins like *Ma*-MxaJ, *Ma*-MxaR, or *Ma*-MxaS, for efficient electron transfer in the methanol oxidation system.

## Materials and Methods

### *Ma*-Cyt*c*_L_ Isolation and Purification

*Ma*-Cyt*c*_L_ was isolated directly from *Methylophaga aminisulfidivorans* MP^T^, which was cultured in mineral salts medium (MSM), an artificial seawater medium, containing 15 mM KH_2_PO_4_, 15 mM (NH_4_)_2_SO_4_, 1.5 mM MgSO_4_•7H_2_O, 0.5 M NaCl, 7.5 μM FeSO_4_•7H_2_O, pH 7.0 supplemented with 1% (v/v) of methanol at 303 K under aerobic conditions for three days as described previously [14, 15]. The cells were pelleted by centrifugation at 5,000 ×*g* and resuspended in standard buffer (40 mM Tris-Cl, 50 mM NaCl; pH 8.0) supplemented with DNase I and lysozyme. The cells were then disrupted by sonication and centrifuged at 35,000 ×*g* for 1 h to obtain a homogenous soluble fraction. The acidic reddish brown-colored *Ma*-Cyt*c*_L_ protein fraction was isolated from the soluble supernatant through anion-exchange and size exclusion chromatography by utilizing its unique properties (Theoretical MW 21,590 Da; pI 5.05). The purified *Ma*-Cyt*c*_L_ was loaded on to a pre-calibrated size- exclusion column HiLoad™ 16/600 Superdex™ 200 pg (GE Healthcare, USA) with final buffer (20 mM Tris-Cl, pH 7.4, 50 mM NaCl). The peak corresponding to monomeric *Ma*-Cyt*c*_L_ (~18 kDa) was collected, pooled together, and concentrated using Centricon (cut-off 10 kDa) (Millipore, USA) for crystallographic and spectrophotometric analysis. The determination of the oligomeric state of *Ma*-Cyt*c*_L_ was performed using standard proteins such as conalbumin (75 kDa), ovalbumin (44 kDa), carbonic anhydrase (29 kDa), and ribonuclease A (13.7 kDa).

### Spectrophotometric Analysis

Spectrophotometric analysis was performed using the pyridine hemochrome assay, as described previously [22]. The reaction mixture was prepared by mixing 0.5 ml of 10 μM purified *Ma*-Cyt*c*_L_ with 0.5 ml pyridine solution (40% v/v pyridine, 0.2 M NaOH) in a 1 cm path length quartz cuvette. The reaction mixture was treated with 500 μM potassium ferricyanide to obtain the oxidized spectrum through a wavelength scan between 350 nm and 650 nm using a spectrophotometer (Biochrom, Libra S22, UK). Subsequently, the oxidized cytochrome reaction mixture was treated with 10 μl of sodium dithionite solution (0.1 M sodium dithionite, 0.5 M NaOH) for the reduction spectrum. The difference in spectra between the reduced and oxidized states was investigated to determine the α, β, and Soret band wavelengths.

### Crystallization

The initial crystallization screening was performed using the hanging drop vapor diffusion method. Each drop, consisting of 1 μl *Ma*-Cyt*c*_L_ (12 mg/ml) and 1 μl of well solution, was incubated at 293 K. Crystallization conditions were extensively examined using commercial screens including including Index, SaltRx, PEG/Ion, PEG/Ion 2, Crystal Screen, Crystal Screen 2, Crystal screen Lite (Hampton Research, USA) and Wizard Screens I, II, III, and IV (Emerald BioStructures Products, USA). The red-colored *Ma*-Cyt*c*_L_ crystals appeared in the wizard screen III- 21 (800 mM sodium phosphate monobasic, 100 mM HEPES/sodium hydroxide, pH 7.5) and Index 74 (0.2 M lithium sulfate monohydrate, 0.1 M Bis-Tris, pH 5.5, PEG 3350 25%) within 21 days. The crystals were then soaked in cryo-solution containing mother liquor reservoir solution with 20% glycerol for 20 s and flash cooled in liquid nitrogen. Of all the crystal conditions described above, wizard screen III-21 produced crystals that diffracted at a high resolution.

### Diffraction Experiment and Structure Determination

The cryoprotected crystals were mounted in a cryogenic N2-gas stream (100 K) during diffraction. X-ray diffraction data for *Ma*-Cyt*c*_L_ crystal were collected from beamline 7A at the Pohang Light source (Korea) using an ADSC Q270 detector with an oscillation of 1.0° and 1 s exposure per frame over a 360° range at a peak wavelength of 0.97934 Å. The crystal from the above condition diffracted to the highest resolution of 2.13 Å, belonging to the monoclinic space group P21. The diffraction data were processed and scaled with *HKL*-2000 (HKL Research Inc., USA). The diffraction statistics are listed in Table 1. Molecular replacement using Phaser [23] of Phenix suite was attempted with the structures of cytochrome *c*_L_ from *Methylobacterium extorquens* (*Me*-Cyt*c*_L_; PDB:2C8S) [7] and *Hyphomicrobium denitrificans* (*Hd*-Cyt*c*_L_; PDB: 2D0W) [6] as model structures, which had sequence identities of 55% and 48%, respectively. Although both the structures failed to provide an initial phase in the first attempt, the molecular replacement (MR) finally worked when the first 32 residues corresponding to the signal peptide were cleaved off from the reference model (PDB: 2D0W). The asymmetric unit contained four molecules with a Matthews coefficient of 2.06 (Å3 Da-1) and a solvent content of 40.38% (Table 1). We noticed a vast region of an unmodeled *2F_o_-F_c_* map, which was incorporated with heme *c*, whose vinyl group formed a covalent bond with Cys88 and Cys91 of *Ma*-Cyt*c*_L_. The resulting model was built manually using *Coot* [24, 25] followed by refinement with *REFMAC5 *[26] and *phenix.refine* [27, 28], which provided a final R_work_/R_free_ value of 14.8%/20.7%. The final quality of the model was examined using MolProbity [29] and uploaded in the PDB database with the accession number 7C90.

### Inductively Coupled Plasma Mass Spectrometry

The metal content of the purified *Ma*-Cyt*c*_L_ holoenzyme was determined using inductively coupled mass spectrometry (ICP-MS) (Model: VGTQ-PQExcel, UK). The protein sample was dialyzed extensively against a buffer containing 20 mM Tris-Cl (pH 7.5) to ensure the removal of loosely bound metal ions. The holoenzyme was digested using nitric acid (final nitric acid concentration ~2-3%), following which the sample was boiled at 90°C for 10 mins. The digested sample was analyzed using ICP-MS for the determination of the metal content of the protein.

## Results and Discussion

### Primary Structure Analysis and Oligomeric State Determination

Analysis of the *Methylophaga aminisulfidivorans* MP^T^ (*Ma*MP^T^) genome by the NCBI server suggested that the *mxaG* gene (582 bp) encoded the cytochrome *c*_L_ (*Ma*-Cyt*c*_L_) protein, which had 194 amino acids and a calculated molecular mass of 21,590 Da. BLASTP searches indicated that the deduced amino acid sequence of the *mxaG* gene had pronounced sequence identity to those of the Cyt*c*_L_ genes in the genome sequences of *Methylobacterium Extorquens* (55% identity), *Paracoccus denitrificans* (53% identity), and *Hyphomicrobium denitrificans* (49% identity). As shown in Fig. 1A, the alignments of the deduced amino acid sequences of *Ma*-Cyt*c*_L_ protein and its homologs revealed that their protein sequences exhibited a highly conserved CXXCH heme-binding motif (i.e., ^88^CSGCH^92^ in *M. aminisulfidivorans* MP^T^) (boxed in Fig. 1A). Additionally, the histidine (H92) and methionine (M132) residues that bind to the Fe atom of heme, generally termed as “axial ligands”, were conserved (green square in Fig. 1A). These observations suggest that the protein encoded by the *mxaG* gene contains a heme- binding motif, as found in other Cyt*c*_L_ homologs.

Further analysis of the amino acid sequence indicated that the first thirty-two residues were predicted to be a signal peptide (using SignalP 5.0 webserver) [30], which transports the protein to the periplasmic space. After reaching the periplasmic space, the signal peptide is cleaved by endopeptidases [31]. Interestingly, the residues involved in the formation of the disulfide bridge at the C-terminus of the protein, as seen in *Me*-Cyt*c*_L_ and *Hd*-Cyt*c*_L_, were absent in *Ma*-Cyt*c*_L_ and *Pd*-Cyt*c*_L_ (orange triangle in Fig. 1A). These similarities in the critical residues between *Ma*-Cyt*c*_L_ and *Pd*-Cyt*c*_L_ and the absence of the C-terminal disulfide cysteine residues indicate that both proteins might possess similar structural features and biological functions in the organism.

**Fig. 2:**
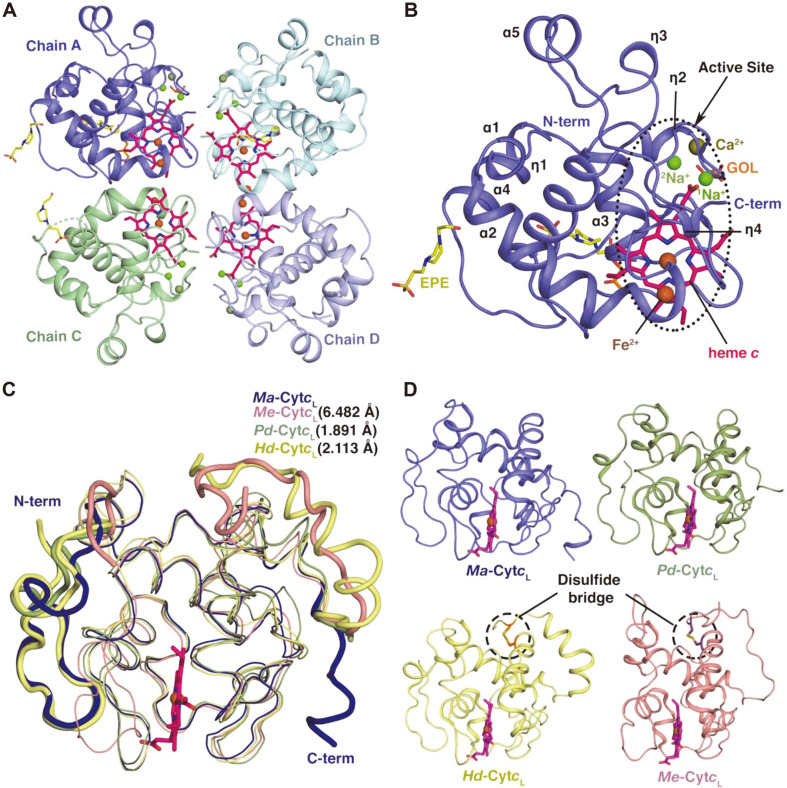
Crystal structure of *Ma*-Cyt*c*L. (**A**) The asymmetric unit (ASU) having four units (shown in ribbon, colored differently as chain A, B, C and D) of *Ma*-Cyt*c*L arranged orderly, with their active sites containing heme *c* (Magenta) facing each other. Each subunit included 2 Na+ (green), 1 Ca2+ (deep olive green), 1 Fe2+ (brown), HEPES (EPE) (yellow) and glycerol (GOL) (orange). (**B**) *Ma*-Cyt*c*L monomer. Both the N- and C- terminal ends are marked and the structural elements are numbered from the N- to the C- terminus. The active site is shown by a dotted oval ring in the monomer. The active site contains heme *c* (Magenta) bound to it. Each monomer has three different metal ions bound to it. (**C**) The monomeric structure of *Ma*-Cyt*c*L was aligned with its structurally close homologs (*Me*-Cyt*c*L PDB:2C8S, *Pd*-Cyt*c*L PDB:2GC4 and *Hd*-Cyt*c*L PDB:2D0W), with the RMSD values mentioned near the alignment. The deviations at the N- and C- terminal ends are shown as tubes, while the core structural elements are shown as lines. (**D**) The structures of homologs are individually shown in the same orientation as C. The bottom panel shows the structures of *Hd*-Cyt*c*L and *Me*-Cyt*c*L that contain the C-terminal disulfide bridge holding the C- terminal end rigid. In contrast, the C-terminal end of *Ma*-Cyt*c*L is flexible.

*Ma*-Cyt*c*_L_ was directly isolated from *Ma*MP^T^ for crystallization by the overexpression of the *mox* operon in media containing 1% methanol. The crystal contains four *Ma*-Cyt*c*_L_ molecules in the asymmetric unit (ASU), in which the active sites (heme-binding pocket) from each subunit face each other (Fig. 2A). Although it appears to be a tetramer in the asymmetric unit, *Ma*-Cyt*c*_L_ likely functions as a monomer in the cell. This hypothesis is based on the following crystallographic facts, which reflect its plausible behavior in solution- a) a relatively small buried surface area (670.3 Å^2^), b) the presence of few interfacial interactions, and c) the low conservation of the residues at the interface. Hence, the ordered arrangement of the four molecules in ASU appears to be due to crystal packing. Furthermore, the size-exclusion chromatography (SEC) analysis revealed that *Ma*-Cyt*c*_L_ elutes with an approximate monomeric molecular weight of 17,648 Da (Fig. 1B), which is consistent with the results of the SDS- PAGE (Fig. 1C), rather than with its homo-tetrameric molecular weight. This reiterates that *Ma*-Cyt*c*_L_ may function as a monomer within the cells, with the signal peptide (32 amino acids) cleaved off by internal bacterial peptidases [31].

### Crystal Structure of *Ma*-Cyt*c*_L_

Each monomer of *Ma*-Cyt*c*_L_ (Fig. 2B) contains a heme cofactor covalently bound to the heme-binding region and coordinated by a metal ion, Fe(III) in heme [32]. In addition, each subunit had one HEPES (EPE) and one glycerol (GOL) molecule bound at the protein surface, which were present in the cryo-solution (see Materials and Methods) (Fig. 2B). Interestingly, three additional metal ions, Na^+^, Fe^2+^, and Ca^2+^, were also found in the subunit, with the Na^+^-binding site coinciding with the metal-binding sites in other structures [6, 7, 21] . Meanwhile, the Ca^2+^ and Fe^2+^ sites are unique to *Ma*-Cyt*c*_L_, which may have originated from supplementation in the seawater medium (MSM).

One hundred and fifty-one amino acid residues (Q33 to K183) out of 194 were assigned to the structure of the *Ma*-Cyt*c*_L_ monomer (Fig. 2B), leaving the last eleven residues (E184 to H194) unrefined at the C-terminus. The structure of *Ma*-Cyt*c*_L_ contains a typical cytochrome *c* fold consisting of six α-helices (Fig. 2B; α1-α6) and four 3_10_ helices (η1-η4). Among the structural elements, four helices (α3, α4, α5, and η2), comprising the CXXCH motif, envelop the heme, as found in other cytochrome *c*_L_ homologs (Fig. 2C). In addition, the core region (E57 to N172) of *Ma*-Cyt*c*_L_ aligns well with those of homologs from methylotrophs, with RMSD values of 0.643 Å (line representation in Fig. 2C). However, the overall structure of *Ma*-CytcL deviates from those of homologs, including *Me*-Cyt*c*_L_, *Pd*-Cyt*c*_L_, and *Hd*-Cyt*c*_L_ (Fig. 2C), with RMSD values of 6.426 Å, 1.891 Å, and 2.113 Å, respectively, possibly as a result of an aberration at the N- and C- termini (tube representation for the ends in Fig. 2C). Of the two regions affecting the deviation, the flexibility in the C-terminal end of *Ma*-CytcL seems to be largely responsible for the overall high RMSD values between the homologs (Figs. 2C and 2D). This is because all the C-terminal residues (over N172 of *Ma*-CytcL) of the homologs appear clearly ordered in their crystal structures with a disulfide bond between C (167, 165) at the C-terminus and C (53, 47) in α2, respectively, for *Me*-Cyt*c*_L_ and *Hd*- Cyt*c*_L_ [6, 7] (Fig. 2D). This disulfide bond provides stability to the C-terminal ends in *Me*-Cyt*c*_L_ and *Hd*-Cyt*c*_L_ [6, 7]. However, the corresponding residues in *Ma*-CytcL were replaced with A76 in α2 and T192 at the C-terminus (Figs. 1A and 2D), resulting in a disordered loop at the C-terminus.

**Fig. 3:**
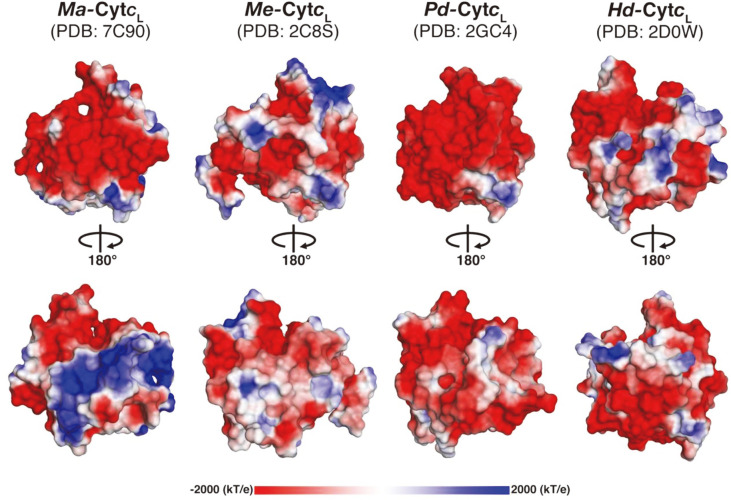
Electrostatic potential comparison between *Ma*-Cyt*c*L and its close homologs. The electrostatic distribution (blue to red corresponds to positive charge +2kT/e to negative charge -2 kT/e) on the surface of *Ma*-Cyt*c*L and its homologs (*Me*-Cyt*c*L, *Pd*-Cyt*c*L, and *Hd*-Cyt*c*L), with the same orientation as Fig. 2B. The positively charged patch in *Ma*-Cyt*c*L is clearly visible compared to the other homologs which are predominantly acidic. The calculation was performed using the APBS 2.1 suite of PyMOL. The protein molecule was rotated 180° along the vertical axis, in order to show the electrostatic potential around the protein molecule.

Despite the structural fold similarities to other homologs (Figs. 2C and 2D), the structure of *Ma*-Cyt*c*_L_ shows an electrostatic potential distribution which is distinctive from those of its homologs (Fig. 3). While negative charge is distributed throughout the protein molecule of the homologs, *the Ma*-Cyt*c*_L_ surface has a sizable positively charged patch, formed by three structural elements-one end of the N-terminus (Q33 to D46), the helix α4 (P112 to Y125), and the loop (G126 to Y136) (Fig. 3). In these regions, basic residues including R37, K52, K113, N116, K118, R129, Q135, and N137 dominate in *Ma*-Cyt*c*_L_, while acidic amino acids occur in most Cyt*c*_L_ from other methylotrophic bacteria (Fig. 1A). Although the biological relevance of the unique surface charge is not clearly understood, the region may participate in the protein-protein interaction for methanol oxidation in this bacterium. Interestingly, it has been reported that the corresponding region of Cyt*c*_L_ (*Pd*-Cyt*c*_L_) from *P. denitrificans* interacts with an adapter protein, amicyanin, which creates a bridge between methylamine dehydrogenase (MaDH) and *Pd*-Cyt*c*_L_ to facilitate the formation of the MaDH-Amicyanin-Cyt*c*_L_ complex (PDB: 1MG2) and electron transfer *via* electrostatic interaction during methylamine oxidation [21]. Similarly, this unique positive binding surface of *Ma*-Cyt*c*_L_ may provide a base for the adapter protein, which can link methanol dehydrogenase to Cyt*c*_L_ (*Ma*-Cyt*c*_L_). Although the adapter protein entity has not yet been identified, one of the *mox* operon proteins, such as MxaJ or MxaS or MxaR, may play a role in the electron transfer during methanol oxidation.

### Heme Coordination at Active Site

**Fig. 4:**
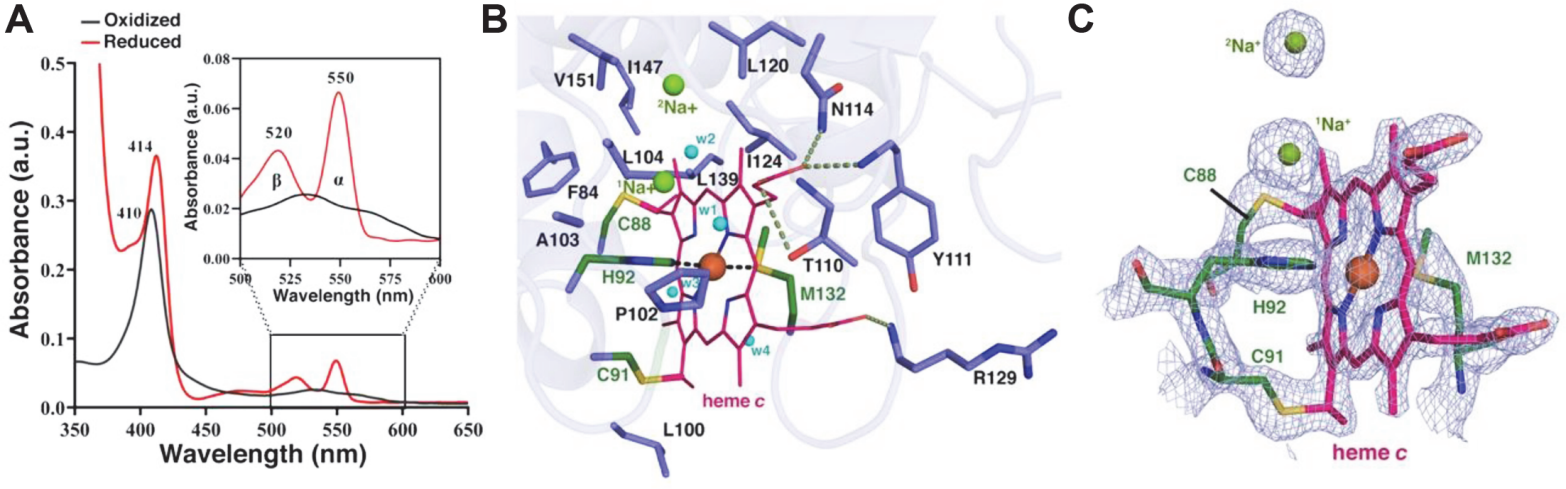
Heme coordination at *Ma*-Cyt*c*L active site. (**A**) UV-Vis absorbance spectrum of the pyridine hemochrome of *Ma*-Cyt*c*L. The red trace shows spectra after reduction with sodium dithionite, and the black trace shows spectra after oxidation with potassium ferricyanide. The α and β peaks corresponding to 550 nm and 520 nm indicate that the species of heme is of the *c* type. (**B**) The heme coordination can be visualized as three layers: the conserved heme binding residues along with axial ligands (green) that form covalent bonds (black dash line) to stabilize heme; the residues that offer hydrogen bonds (green dashed line); and the hydrophobic pocket (blue) around heme (magenta). All residues are shown as sticks. The water molecules (w1-w4) that stabilize heme with their hydrogen bonds are shown as blue spheres. (**C**) The 2F_o_-F_c_ map (light blue color), contoured to 1.5 s around the heme, shows the exact coordination of the heme-binding residues (C91 and C88) forming a disulfide bridge with a vinyl group of heme. The maps around the axial ligands (H92 and M132) and the metal ions ^1^Na^+^ and ^2^Na^+^ are shown.

As stated above, the *Ma*-Cyt*c*_L_ structure contains a heme molecule coordinated by surrounding α-helices, a loop between α3 and η2 (G93 to Y111), and the CXXCH motif in α3 (Figs. 2B and 4B). Although the type of heme molecule involved in methanol oxidation seems to be classified as the *c* type, in-depth biochemical studies on the types of heme and cytochrome from *Ma*MP^T^ have not yet been conducted. The spectroscopic study showed the typical hemochromagen spectral pattern of the *c* type of heme called the soret band, in which the band at 410 nm in the oxidized state shifts to 413 nm when reduced (Fig. 4A). Besides, a spectrum separation to 520 and 550 nm occurred within 500-600 nm, depending on the oxido-reduction state of heme, which is also unique to heme *c* [33] (Fig. 4A inlet). In addition, the calculated pI value of *Ma*-Cyt fell to ~5.1, which indicates that the Cyt protein belongs to the L (low pI) subtype of cytochrome [5]. Therefore, we concluded that the cytochrome from *Ma*MP^T^ could be classified as Cyt*c*_L_, similar to other homologs from methanotrophs.

The active site of the enzyme in the crystal structure (dotted oval ring in Fig. 2B) contains heme *c*. The heme coordination with *Ma*-Cyt*c*_L_ can be visualized as three-layered structure (Figs. 4B and 4C) from the heme to the surface- the strong covalent bond interactions, including the residues of the heme-binding motif (^88^CXXCH^92^) (green colored in Figs. 4B and 4C); a layer of hydrogen bonds; and a layer of the hydrophobic pocket. The heme not only covalently bonds with C88 and C91 *via* a vinyl group, but also interacts with ferric ion through the two pyrrole rings on heme *c*, indicating that *Ma*-Cyt*c*_L_ contains the characteristic low-spin Fe at the center of heme *c* [5] (Figs. 4B and 4C). The iron atom is further coordinated by the axial ligands, including H92 and M132, as in typical cytochromes (Figs. 1A, 1B, and 4C). Besides the heme-binding motif, the heme interacts with surrounding residues mainly through hydrogen bonds, wherein the carbonyl oxygen atoms of heme form hydrogen bonds with nearby residues- O1A with N114 and Y111, O1B with R129 and Y111, and O2A with T110- of the protein. In the next layer, the hydrophobic pocket surrounds the above residues and the heme-binding motif, which consists of F84, A87, L100 P102, L104, L120, I124, L139, I147, and V151 from four helices and loop (α2, α3, α4, α5, and loop between α3 and η2) (Fig. 4B). In this regard, the three structural layers seem to stabilize heme *c* inside the cytochrome molecule for efficient electron transfer [34] and protect the active site from the bulk solvent [7].

**Fig. 5:**
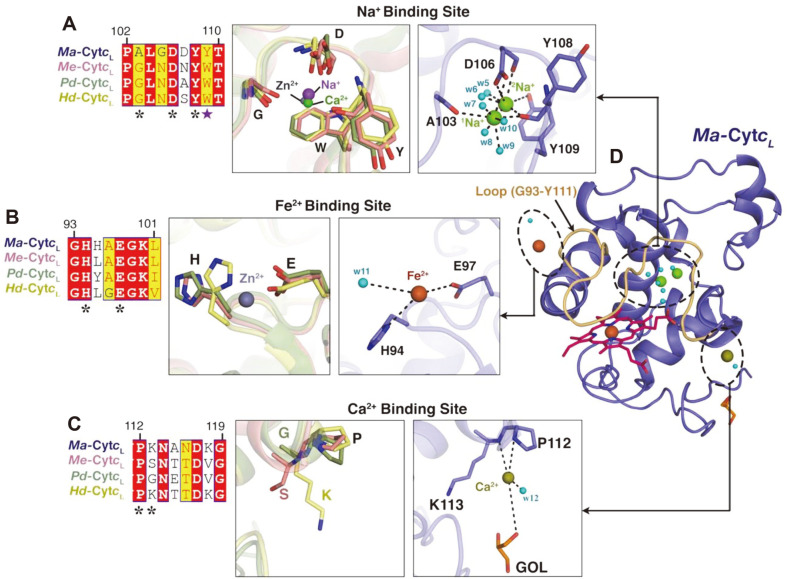
Metal-binding sites present in *Ma*-Cyt*c*L. The crystal structure of the *Ma*-Cyt*c*L monomer contains three metal binding sites- Na+, Fe2+, and Ca2+- shown enlarged in the ‘Right’ of panels (**A**), (**B**), and (**C**). The conservation of the metal- coordinating residues is shown in sequence alignment with their close homologs in ‘Left’ of each panel (**A**), (**B**), and (**C**). The star sign shows the residues that coordinate with the metal, and their conservation can be compared with the sequence of other homologs. Their corresponding sites in other terrestrial homologs are mentioned in 'Middle' of each panel (**A**), (**B**), and (**C**). Metal ions and water molecules are shown as coordination spheres, while the residues are shown as a stick model. The metal ions that stabilize the loop G93 to Y111 are colored light orange in the cartoon representation.

Other than Fe in the heme, the presence of an additional metal ion closer to the heme propionate group is a characteristic feature of the *c*_L_ group of Cyt*c* [6, 7, 21]. Interestingly, in contrast to other Cyt*c* homologs, Cyt*c*_L_ from methylotrophs contained one of either Ca^2+^, Zn^2+^, or Na^+^ metal ions, which were coordinated with glycine, aspartic acid, and tyrosine (Ca^2+^ in *Me*-Cyt*c*_L_, Zn^2+^ in *Hd*-Cyt*c*_L_, and Na^+^ in *Pd*-Cyt*c*_L_; in Fig. 5A Middle) [6, 7, 21]. However, *Ma*-Cyt*c*_L_ contains two Na^+^ ions (^1^Na^+^ and ^2^Na^+^) mainly surrounded by the loop between α3 and η2, in which a few residues form coordination bonds with the heme molecule (Figs. 1A and 5A right). The two Na ions were located near to each other (~3.9 Å) and clearly distinguished in the electron density map (Figs. 2A, 5A, and S3). The first Na ion (^1^Na^+^) corresponds to the metal-binding sites of homologs, wherein the metal ion is hydrogen-bonded to A103, D106, and Y108 and water molecules (w7-w10) (Fig. 5A, right). The presence of a second Na ion (^2^Na^+^) in *Ma*-Cyt*c*_L_ could be explained by the fact that the highly conserved tryptophan near this metal-binding site in the Cyt*c*_L_ homologs was replaced with tyrosine Y109 in *Ma*-Cyt*c*_L_ (Figs 1A; marked purple star and 5A). This replacement might release steric hindrance to provide room for the entry of the second Na ion, which, in turn, stabilizes the coordination *via* interaction with Y109, D106, and Y108 (Fig. 5A right). It has been proposed that the Na^+^- binding site in the _L_ group of cytochrome *c* functions similar to the arginine residue in other subtypes of cytochrome c (such as in Arg39 in tuna) [35], whose side chain coincides with the Na^+^ site and interacts with the ionized propionate of heme to contribute a high redox potential to cytochrome *c* [7]. Therefore, the two Na metal ions in *Ma*-Cyt*c*_L_ may enhance the degree of the overall redox potential by contributing an additional positive charge to the heme propionate compared to other terrestrial homologs. Furthermore, these unique structural features of *Ma*-Cyt*c*_L_ supposedly play a role in the stability of the backbone of the loop (P102 to T110) (Fig. 5D), which can help the loop present proximal to heme cofactor. However, the precise function of the loop connecting with the ions needs further investigation.

### Novel Metal-binding Sites in *Ma*-Cyt*c*_L_

The structure of *Ma*-Cyt*c*_L_ had a novel Na^+^- binding site near the heme, which has not been observed in other Cyt*c*_L_ structures. Interestingly, the 2F_o_-F_c_ electron density of each subunit showed unique, distinguishable peak regions, which are likely to correspond to two additional metal-binding sites. The metals for each site were selected based on least B-factor and rejections by the refinement on a trial-and-error basis (Fig. S3), and further augmented by ICP-MS analysis, whose maps were finally designated as Fe^2+^ and Ca^2+^ in the final model (Fig. 5).

The Fe^2+^ binding site in *Ma*-Cyt*c*_L_ is coordinated by typical metal-binding residues H94 and E97 (Figs. 1A and 5B right) and further stabilized by hydrogen bonds with a nearby water molecule (w11) (Fig. 5B; right). Although the metal-binding residues were highly conserved in all Cyt*c*_L_ homologs (Fig. 5B; left), metals were found only in *Hd*-Cyt*c*_L_ with zinc [6] and *Ma*-CytCL with Fe, respectively. Although rare, this metal site is likely a crucial part of the protein, and not a random crystal artifact, due to the following reasons; a) the coordination by typical metal- binding residues Asp and Glu, and b) the absence of Fe^2+^ in the crystal solution. Interestingly, Fe^2+^ interacts with the two residues H94 and E97 on the loop (G93 to Y111) to stabilize the N-terminal portion of the loop (orange in Fig. 5D). As mentioned earlier, several residues from the middle of the loop indirectly stabilize the heme through coordination with the two Na ions. Therefore, together with the extra Na ions, the additional Fe^2+^ may play a role in the proper positioning and stabilization of the heme inside the molecule. In addition to the Fe-mediated loop stabilization (G93 to Y111) (Fig. 5D), the metal is likely to provide *Ma*-Cyt*c*_L_ with a negatively charged base for charge-charge interactions with the binding partner.

The unique Ca^2+^-binding site positioned at the C-terminus of the loop (G93 to Y111) (orange in Fig. 5D) of *Ma*-Cyt*c*_L_, is absent in other terrestrial homologs. This metal site is coordinated by atypical metal-binding residues such as the backbone carbonyl oxygen of K113 (η2), the nitrogen atom of proline P112 (η2), the oxygen atoms from the water molecule (w12), and the glycerol molecule (GOL) located near the metal (Fig. 5C; right). Although it is possible that this metal is loosely bound to the surface of the protein, it is still an integral part of the protein, which is backed by our ICP-MS data. In accordance with this, the significance of the proline residues in the calcium-binding sites in several other proteins such as thermolysin (PDB:6D5P), staphylococcal nuclease (PDB:1AEX), and concanavalin A (PDB:6AHG), was investigated [36]. Interestingly, in all these proteins, calcium is coordinated by the nitrogen of proline and the carbonyl backbone of the adjacent residue. Indeed, this typical binding conformation reaffirms the unique calcium-binding site in *Ma*-Cyt*c*_L_. With respect to the functional significance of calcium ions, it has been known to contribute to the thermostability in thermolysin [37] and several other calcium-binding proteins. Hence, it is possible that the presence of this calcium site, which is unique in marine *Ma*-Cyt*c*_L_, might enhance the stability of the protein compared to its terrestrial homologs.

The presence of three unique metal binding (Na^+^, Fe^2+^, and Ca^2+^ in Fig. 5) sites in each monomer of *Ma*-Cyt*c*_L_ could be clearly identified based on the electron density map (Fig. S3), and all these metals might play a unique role with respect to their position. Interestingly, all the metal-binding sites (Na^+^, Fe^2+^, and Ca^2+^) appeared to stabilize the loop (G93 to Y111) located at the ceiling of heme (Fig. 5D). Although the precise function of this loop is not clear, the highly conserved residues and their occurrence in all cytochromes point to a significant biological role. The stabilization of the loop in *Me*-Cyt*c*_L_ with the aid of metal binding at different sites along the loop might enhance the steadiness of the loop, a unique feature not seen in terrestrial homologs.

### Electron Transfer from MDH to Cyt*c*_L_

The precise electron transfer mechanism between MDH and Cyt*c*_L_ remains unknown in methylotrophs, primarily because the roles of the gene products, including MxaJ, MxaR, and MxaS, of the *mox* operon have not yet been fully elucidated. Hence, several questions regarding the direct electron transfer from MDH to Cyt*c*_L_ remain unanswered. The intact complex of MDH with Cyt*c*_L_ has not been isolated in the presence of methanol, which may suggest that the interaction between the proteins is not strong enough to hold the two proteins together. The *in silico* docking of *the Hd*MDH-Cyt*c*_L_ complex model [6] showed an incongruous distance for the least required direct electron jump (within ~20 Å) between their active site cofactors (from PQQ in MDH to heme in Cyt*c*_L_). In addition, the electron transfer between MDH and Cyt*c*_L_ was reported to be inhibited by sodium chloride [6], indicating that electron transfer could be hindered in a high salinity marine environment. These observations led us to hypothesize that electron transfer seems to be efficiently mediated by an adapter protein *via* protein-protein interaction between MDH and Cyt*c*_L_. Possibly, the protein, which plays the role of an adapter or mediator, could be found in the *mox* operon components. Among the operon components, MxaJ is the most likely soluble periplasmic protein that could facilitate the electron transfer by bridging and adopting a suitable orientation for a proper contact requiring an electron jump (less than ~14 Å) with allowable distance [11, 12]. Although MxaJ of terrestrial bacteria has not been studied [38], its structure and plausible role with respect to methanol oxidation in aquatic methylotrophs was first postulated by our group [12]. Our hypothesis can be further augmented by the known complex structure of Methylamine dehydrogenase (MaDH)-amicyanin-CytcL from the soil bacterium *Paracoccus denitrificans* (PDB: 1MG2), in which the copper-containing amicyanin plays the role of an adapter protein during the electron transfer between MaDH and Cyt*c*_L_ [21]. Indeed, m*auC,* encoding amicyanin, is a member of the *mau* operon genes [39]. Thus, *Ma*-MxaJ may be able to facilitate the electron transfer as an adapter protein between *Ma*-MDH and *Ma*-Cyt*c*_L_. In this regard, *Ma*-MxaJ may provide a platform for the interactions between *Ma*-MDH and *Ma*-Cyt*c*_L_ (Fig. S2). *Ma*-MDH has a patched acidic region, and *Ma*-Cyt*c*_L_ has a basic region at the N-terminus (Figs. 3, S2A, and S2C). Remarkably, *Ma*-MxaJ presents two electrostatically distinctive regions, including a negatively charged central cavity [12] and the basic loop L8 [12] (Fig. S2B). This electronically bi-functional nature of *Ma*-MxaJ likely enables this protein to act as a scaffolder and accept both *Ma*-MDH and *Ma-*CytcL-like amicyanin in *mau* operon. To verify the above hypothesis, biochemical and kinetic studies regarding the roles of each component for electron transfer are currently in progress. However, the possibility that other components in the *mox* operon, including *Ma*-MxaS and *Ma*-MxaR, may function as an adapter protein, cannot be ruled out.

## Summary

We determined the crystal structure of the soluble electron receiver of the *mox* operon, *Ma*-Cyt*c*_L_, from aquatic methylotroph *Ma*MP^T^. Despite sharing high sequence identity with other terrestrial Cyt*c*_L_ homologs, the structure reveals some unique features specific only to *Ma*-Cyt*c*_L_. Apart from Fe of heme, *Ma*-Cyt*c*_L_ contains three different metal-binding sites (Na^+^, Fe^2+^, and Ca^2+^), which play an important role in stabilizing heme coordination and the loop (G93-Y111). Furthermore, an additional Na ion in the active site provides an increased positive charge to compensate for heme propionate, which might enhance the overall redox potential of *Ma*-Cyt*c*_L_. Finally, in order to increase the affinity with its binding partner, the N- and C-terminal ends of *Ma*-Cyt*c*_L_ offer positive and negative charged surfaces, respectively, which might increase potential contact points with the binding partner. In conclusion, these structural findings lead us to postulate that *Ma*-Cyt*c*_L_ might bind with *Ma*-MxaJ, which contains a bipolar surface and hence may act as an adapter protein, for easy electron jumps between *Ma*-MDH and *Ma*- Cyt*c*_L_ during methanol oxidation. Based on our proposal in terms of proteins in the the methanol oxidation process, the successful reconstitution of the ternary complex in vitro would render a mass production of metabolic intermediates such as formic acid and formaldehyde, which reside in the center of the useful organic compound in the pharmaceutical industry. Moreover, the in vitro interconnection of the active methanol oxidation system with methane oxidation complex from aquatic methanotrophs could be applied to decrease the notorious greenhouse gas, methane.

## Supplemental Materials



Supplementary data for this paper are available on-line only at http://jmb.or.kr.

## Figures and Tables

**Table 1:** Crystallographic data collection statistics for *Ma*-Cyt*c*_L_

Diffraction statistics	*Ma*-Cyt*c*_L_ (PDB: 7C90)
Beamline	PLS-7A
Wavelength (Å)	0.97934
Temperature (K)	100
Space group	P2_1_
Cell parameters	
*a*, *b*, *c* (Å)	61.76, 76.06, 66.44
*α*, *β*, *γ* (^o^)	90.00, 106.84, 90.00
Data resolution (Å)	50.00-2.13 (2.17-2.13)
Completeness (%)	99.9 (97.8)
Redundancy	7.2 (7.4)
Total reflection	237,733
Unique reflections	32,914
R_merge_^a^ (%)	10.3 (63.0)
Average I/s	4.9 (3.0)
Matthew’s coefficient (Å^3^ Da^-1^)	2.06
Solvent content (%)	40.38
No. of chains per asymmetric unit	4
Refinement	
R_work_/R_free_ (%)	15.78/20.95
Protein residues/water	602/284
RMSD	
Angle (°)	1.25
Length (Å)	0.0093
Average B-factors (Å^2^)	42.97
Ramachandran plot	[1]
Most favored regions (%)	96.79
Allowed regions (%)	3.04
Outliers (%)	0.17

Values in parentheses correspond to the highest-resolution shell

^a^R_merge_=S*hk*l S*_i_*|*I_i_*(*hkl*) − <*I*(*hkl*)>|/S*_hkl_* S*_i_* I_*i*_(*hkl*), where *I_i_*(*hkl*) and <*I*(*hkl*)> are the intensity of an individual reflection and the mean value of all measurements of an individual reflections, respectively.
