# Supplementary material for: Crystal Structure of Cytochrome cL from the Aquatic Methylotrophic Bacterium Methylophaga aminisulfidivorans MPT
Source: J Microbiol Biotechnol. 2020 May 20;30(8):1261–71. doi: 10.4014/jmb.2006.06029 (PMC9728263; doi:10.4014/jmb.2006.06029)
Supplement: Supplementary file 1 [file JMB-30-8-1261-supple.pdf]

Supplementary Information

J. Microbiol. Biotechnol.
https://doi.org/10.4014/jmb.2006.06029

Supplementary figures

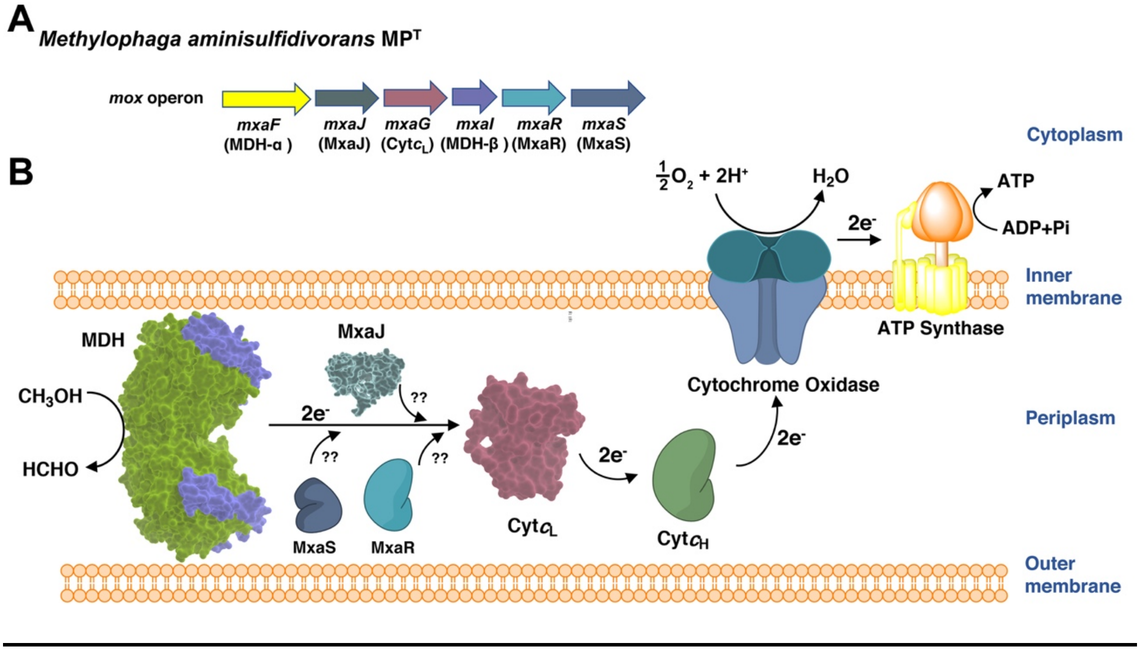

Figure S1: *Mox* operon and Methanol oxidation system in Methylotrophs.

Methylotrophs contain an active *mox* operon encoding the methanol oxidizing proteins. A) The organization of the *mox* operon from *Methylophaga aminisulfidivorans* MP^T, consisting of *mxaFJGIRS*. The proteins encoded by those genes are marked below the name of the genes. B) The sequence of reactions occurring at the periplasm. Firstly, methanol dehydrogenase (MDH), which is a heterotetramer ($\alpha_2\beta2$) (PDB: 5XM3), catalyzes the oxidation of methanol to formaldehyde, utilizing the pyrroloquinoline quinone (PQQ) cofactor present in its active site. The α-subunit is colored green while the β subunit is colored violet. Next, the electron from PQQ of MDH is transferred to the heme of cytochrome c_L (Cytc_L). This electron transfer could be aided by a tertiary protein MxaJ (PDB:5SV6) or MxaS or MxaR, whose role in electron

transfer is unknown. The electron is subsequently transferred to cytochrome c_H (Cytc_H), cytochrome oxidase and finally to ATP synthase which utilizes this electron in the generation of ATP.

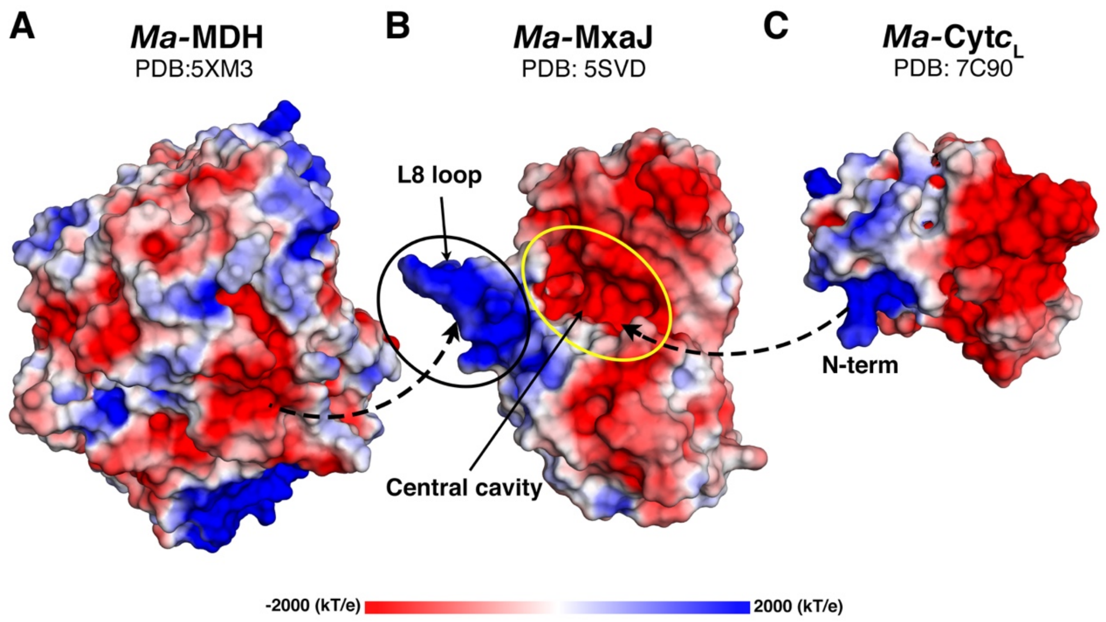

Figure S2: Proposed electron transfer from MDH to Cytc_L

The surface electrostatic potential of the components encoded by the *mox* operon. A) *Ma*-Methanol dehydrogenase (MDH) monomer, B) *Ma*-MxaJ, and C) *Ma*-CytcL, were calculated using the APBS suite of PyMOL. It is postulated that the acidic patch of *Ma*-MDH might interact with the basic loop L8 of *Ma*-MxaJ, while the basic N-terminal end of *Ma*-Cytc_L might interact with the acidic central cavity of *Ma*-Cytc_L. *Ma*-MxaJ shows a bipolar surface, making it a likely candidate as an adapter protein to interact with both *Ma*-MDH and *Ma*-Cytc_L, which might aid in the formation of a ternary complex between them.

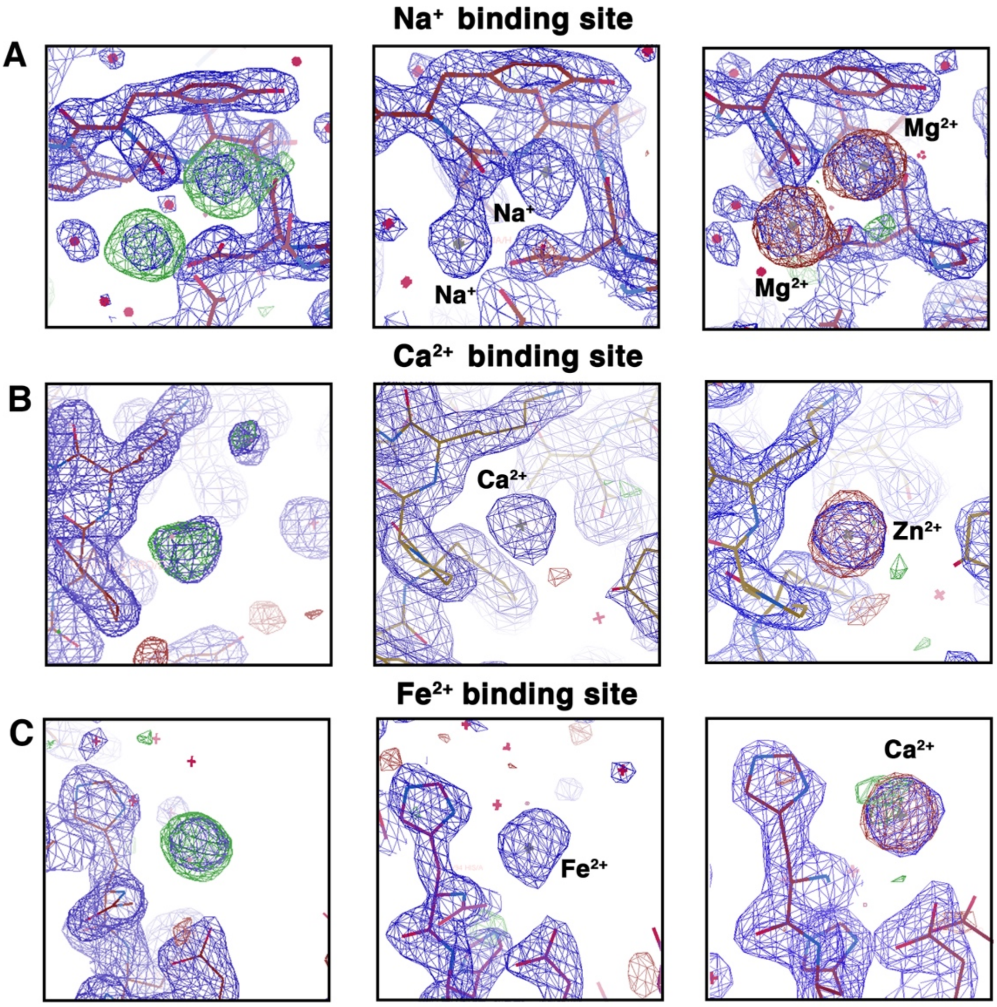

Figure S3: Selection of metals for metal binding sites.

The Electron density *Fo-Fc* (violet) maps contoured to 1σ at the corresponding (A) Na^{+}, (B) Ca^{2+} and (C) Fe^{2+} metal binding sites are shown. The left panel shows the *OMIT* map corresponding to metal sites with the *2Fo-Fc* map shown in green. The middle panel shows the

acceptance of metal upon refinement with no positive or negative peaks. The right panel shows the negative peaks when wrong metals are placed in those sites. The metal in the sites were finalized by the trial-and-error based the rejections and ICP-MS analyses.
